# Evaluation of an Antisense Oligonucleotide Targeting CAG Repeats: A Patient-Customized Therapy Study for Huntington’s Disease

**DOI:** 10.3390/life14121607

**Published:** 2024-12-04

**Authors:** Sergio Adrian Ocampo-Ortega, Vivany Maydel Sierra-Sanchez, Citlali Margarita Blancas-Napoles, Asdrúbal González-Carteño, Elvia Mera-Jiménez, Martha Edith Macías-Pérez, Adriana Hernandez-Guerra, Rodrigo Romero-Nava, Fengyang Huang, Enrique Hong, Santiago Villafaña

**Affiliations:** 1 Laboratorio de Terapia Génica Experimental, Escuela Superior de Medicina, Instituto Politécnico Nacional, Ciudad de Mexico 11340, Mexico; sergio_ocampo05@hotmail.com (S.A.O.-O.); vivany.s44@gmail.com (V.M.S.-S.); citla91@hotmail.es (C.M.B.-N.); a.gonzalezcarteno@gmail.com (A.G.-C.); ady.hernandezg@gmail.com (A.H.-G.); roloromer@gmail.com (R.R.-N.); 2 Laboratorio de Cultivo Celular, Neurobiología y Conducta, Escuela Superior de Medicina, Instituto Politécnico Nacional, Ciudad de Mexico 11340, Mexico; elviamj@gmail.com (E.M.-J.); marthita_e23@yahoo.com.mx (M.E.M.-P.); 3 Laboratorio de Investigación en Obesidad y Asma, Hospital Infantil de Mexico “Federico Gómez”, Ciudad de Mexico 06720, Mexico; huangfengyang@gmail.com; 4 Departamento de Neurofarmacobiología, Centro de Investigación y de Estudios Avanzados, Ciudad de Mexico 14330, Mexico; enriquehong@hotmail.com

**Keywords:** CAG cluster, Huntington’s disease, Huntingtin, long antisense oligonucleotide

## Abstract

Huntington’s disease is a genetic disorder characterized by progressive neuronal cell damage in some areas of the brain; symptoms are commonly associated with chorea, rigidity and dystonia. The symptoms in Huntington’s Disease are caused by a pathological increase in the number of Cytokine-Adenine-Guanine (CAG) repeats on the first exon of the Huntingtin gene, which causes a protein to have an excessive number of glutamine residues; this alteration leads to a change in the protein’s conformation and function. Therefore, the purpose of this work was to design, synthesize and evaluate an antisense oligonucleotide (ASO; 95 nucleotides) HTT 90-5 directed to the Huntingtin CAG repeats in primary leukocyte culture cells from a patient with Huntington’s Disease; approximately 500,000 leukocytes per well extracted from venous blood were used, to which 100 pMol of ASO were administered, and the expression of Huntingtin was subsequently evaluated at 72 h by RT-PCR. Our results showed that the administration of the HTT 90-5 antisense decreased the expression of Huntingtin mRNA in the primary culture leukocyte cells from our patient. These results suggest that the use of long antisense targeting the CAG Huntingtin cluster may be an option to decrease the expression of Huntingtin and probably could be adjusted depending on the number of CAG repeats in the cluster.

## 1. Introduction

Huntington’s Disease (HD) is a neurodegenerative disease named after Dr. George Huntington, who first published a description of its signs and symptoms in 1872. George Huntington referred to this condition as “hereditary chorea” due to the involuntary movements resembling a dance. HD is a rare, inherited autosomal dominant and neurodegenerative disease that causes the progressive degeneration of nerve cells in the CNS [1]. The disease affects three main domains: cognitive, psychiatric and motor. Cognitively, there is a noticeable reduction in decision-making abilities, particularly those related to goal-directed tasks. Verbal learning and visuospatial skills are also compromised [2]. Psychiatric symptoms such as anxiety, irritability, depression, aggression and psychosis complete the clinical profile [3]. Motor manifestations, associated with the progressive degeneration of the striatum, typically manifest between the ages of 30 and 59 [4,5].

The prevalence of HD is approximately 10 cases per 100,000 people, with a high prevalence in North America, Northwestern Europe, the Middle East and Australia, where estimates range from 5.96 to 13.70 cases per 100,000 people [6,7,8,9]. Although there is no unified classification system, the disease is often categorized by the age of onset and the predominant clinical characteristics. Juvenile-onset HD typically presents before the age of 20 and is characterized by bradykinesia, rigidity and seizures, whereas adult-onset HD often manifests later in life with predominant symptoms of chorea and psychiatric disturbances. This categorization also reflects underlying genetic differences, with juvenile-onset cases often associated with higher numbers of CAG repeats and greater genetic instability. The etiology of the disease is linked to an increase in the number of CAG repeats (greater than 40) in the first exon of the Huntingtin gene, located on chromosome 4 [10]. Under normal conditions, it ranges from 6 to 35 without presenting pathological manifestations; in fact, increased repeats within this range may even enhance cognitive skills. In the general population, most normal Huntingtin alleles contain fewer than 30 CAG repeats, with the distribution peaking between 15 and 25 repeats [11]. However, when the number of repetitions reaches between 36 and 39, the disease manifests with incomplete penetrance, and when it exceeds 40, the penetrance is complete [12,13].

The *Huntingtin gene* (Gene ID: 3064) produces a protein of 3144 amino acids (considering a polyQ extension of 23 residues, NCBI reference NP_002102.4) and a molecular weight of 348 KDa [14]. However, when there is an increase in the number of CAG repeats, this leads to an abnormal extension of glutamines residues at the N-terminal end of the protein, causing toxicity and aggregation [15].

This protein is involved in vesicular transport within secretory and endocytic pathways by interacting with dynein [16], and it also plays an important role in the transport and release pathways of certain neurotrophic factors, such as brain-derived neurotrophic factor (BDNF) [17]. However, in HD, the mutant protein and mRNA forms aggregate and trigger apoptosis, thereby contributing to neurodegeneration [18].

Currently, there are no treatments that can cure the disease or slow the progression. Several therapeutic approaches have been explored to target underlying the genetic cause. Antisense oligonucleotides (ASOs), such Tominersen, have shown efficacy in the reduction in mutant Huntingtin expression, advancing to phases I and III in clinical trials. Similar therapies based on this kind of technology like WVE-120102 are in phases I/II trials, aiming to silence Huntingtin mRNA selectively. Other technologies, such CRISPR/Cas9, have demonstrated the potential for directly modifying CAG repeats expansions in preclinical studies, exploring means for a permanent correction [19,20,21,22].

Personalized gene therapy has shown significant promise in addressing rare genetic disorders, as exemplified by the development of Milasen for Batten disease. This therapy utilized patient-derived fibroblasts and other accessible cell types to evaluate efficacy and safety, demonstrating that alternative cellular models can reliably replicate therapeutic mechanisms when derived from the same individual. These precedents highlight the value of using proxy models to overcome practical challenges, such as the limited availability of CNS tissue [21]. Given the challenges of delivering ASOs to the central nervous system, intranasal administration is emerging as a promising strategy for bypassing the blood–brain barrier and achieving direct brain targeting [23,24,25]. Thus, the objective of this work is to reduce Huntingtin gene expression through an antisense oligonucleotide directed at the CAG repeat cluster in the Huntingtin gene.

## 2. Materials and Methods

### 2.1. Patient Selection

The patient was selected based on clearly defined inclusion criteria, including a confirmed diagnosis of Huntington’s Disease (HD) with >40 CAG repeats in the HTT gene, the ability to tolerate blood draws and the provision of informed consent. The exclusion criteria included severe HD preventing decision-making, active psychosis, confusion, violent behavior, severe depressive states and co-infections with HIV, hepatitis B or hepatitis C. The elimination criteria involved a failure to attend both scheduled study intakes, voluntary withdrawal from the study or the development of depression, anxiety or other psychiatric conditions during the study that would impede continued participation.

### 2.2. Sample Collection and Separation of Leukocytes by Density Gradient

Blood samples (6 mL) were collected via venipuncture from the median cubital vein with a 21 G needle and placed in heparinized tubes. Leukocytes were separated using a double density gradient of Ficoll-Paque Plus (Cytiva, Marlborough, MA, USA) (1.077 g/mL and 1.091 g/mL) in 15 mL Falcon tubes [19], followed by centrifugation at 2000× *g* for 30 min at 4 °C. The upper layer containing leukocytes was collected, and the cells were washed twice with Hanks’ balanced salt solution (HBSS) (Gibco, Thermo Fisher Scientific, Waltham, MA, USA) to remove plasma and Ficoll (Cytiva, Marlborough, MA, USA) residues. The cells were then quantified (1,000,000 cells) and seeded in six-well culture plates with RPMI-1640 medium (Gibco, Thermo Fisher Scientific, Waltham, MA, USA) with serum 10% (Gibco, Thermo Fisher Scientific, Waltham, MA, USA.) bovine fetal (Gibco, Thermo Fisher Scientific, Waltham, MA, USA) and 1% antibiotic (Gibco, Thermo Fisher Scientific, Waltham, MA, USA.). The cells were incubated at 37 °C in 5% CO_2_. The cells were divided into three groups and cultured until reaching 90% confluency:

- Group 1: Leukocytes of patient with Huntington’s Disease (H).
- Group 2: Leukocytes of patient with Huntington’s Disease + Transfection Vehicle (Lipofectamine (Thermo Fisher Scientific, Waltham, MA, USA)) (H + L).
- Group 3: Leukocytes of patients with Huntington’s Disease + Transfection Vehicle (Lipofectamine (Thermo Fisher Scientific, Waltham, MA, USA)) + Long antisense oligonucleotide HTT 90-5 (H + L + A)

After 72 h of treatment with the HTT 90-5 antisense oligonucleotide, mRNA was extracted using the Trizol reagent method (Thermo Fisher Scientific, Waltham, MA, USA), followed by RT-PCR analysis.

### 2.3. Design and Synthesis of the Antisense Targeting the HTT Cluster

The patient has 54 CAG repetitions in the Huntingtin gene, corresponding to a CAG cluster length of 162 nucleotides. The long antisense was designed using the Huntingtin mRNA sequence from the GenBank database. The antisense oligonucleotide was designed with 95 nucleotides; the first 5 nucleotides hybridized upstream of the CAG cluster, and the remaining 90 nucleotides hybridized to the CAG repeats region in the Huntingtin mRNA.

Off-target effects were evaluated using the NCBI BLAST software (Version 2.15.0, National Center for Biotechnology Information (NCBI), Bethesda, MD, USA. Available at: https://blast.ncbi.nlm.nih.gov, accessed on 5 March 2024) The complete sequence did not show significant similarity to other sequences. A table was generated showing different lengths of the antisense and potential off targets. We also performed an analysis of possible consequences of this non-specific hybridization.

Secondary structure prediction of the Huntingtin mRNA was performed using RNAfold software (Version 2.4, ViennaRNA Package, University of Vienna, Vienna, Austria, Available at: http://rna.tbi.univie.ac.at/cgi-bin/RNAWebSuite/RNAfold.cgi, accessed on 16 April 2024) to assess the secondary structure formed by this region and determine whether steric hindrance could impede antisense oligonucleotide hybridization. We used the Version 2.0, i-mRNA software (Available at: http://crdd.osdd.net/raghava/imrna/, accessed on 2 September 2024) to predict the immunomodulatory potential of the antisense oligonucleotides.

The Antisense Oligonucleotide were synthesized using a combination of dT and all other RNA nucleotides. The synthesis process was carried out using the Mermade-8 Bioautomation synthesizer (BioAutomation, Plano, TX, USA), following the manufacturer’s protocol [26], which involved a four-step cycle: unlocking, coupling, capping and oxidation [27]. After synthesis, the sequences were cleaved from the solid support, deprotected using NH4OH solution and desalted. The purified sequences were suspended in RNase-free water and quantified, and the purity was assessed using a Nanophotometer (Implen, Munich, Germany). The oligonucleotides were stored at −70 °C until further use. To validate the correct synthesis and determine the length of the HTT-95 antisense oligonucleotide, we performed electrophoresis on a 2% agarose gel.

### 2.4. Transfection of Sequences to Lymphocytes

For the administration of the antisense HTT 90-5, six microliters of Lipofectamine 2000 (Thermo Fisher Scientific, Waltham, MA, USA) per well of a six-plate were used as a transfection agent following the manufacturer’s recommendations. A working solution of antisense HTT 90-5 was prepared at a final concentration of 100 picomoles per milliliter in Opti-MEM, a serum reduction medium was designed to optimize transfection efficiency. The preparation of the lipofectamine and anti-sense complexes was as follows: The first step was the dilution of the HTT 90-5 anti-sense in 125 mL of Opti-MEM. In parallel, six microliters of Lipofectamine 2000 were diluted in another 125 mL aliquot of Opti-MEM separately. Both solutions were incubated for 10 min at room temperature. Then, the dilutions of Lipofectamine 2000 and antisense were mixed and incubated for 10 min at room temperature. The preparation was added to the lymphocytes, 250 μL of the preparation was added and the cells were incubated under optimal culture conditions (37 °C, 5% CO_2_, 95% humidity) for 72 h to allow for transfection and the expression changes. Cell morphology was monitored. Leukocytes were chosen as a proxy model for this study due to their accessibility and established utility in antisense oligonucleotide evaluations. This approach aligns with personalized therapy precedents, such as the development of Milasen, where patient-derived fibroblasts successfully replicated the therapeutic effects despite not being CNS-specific. Using leukocytes ensures that the genetic background of the patient is preserved, allowing for an accurate evaluation of allele-specific targeting by HTT90-5 [21].

### 2.5. MTT Assay Cell Viability

To assess the viability of THP-1 cells treated with 100 pM of the antisense oligonucleotide HTT 90-5, we performed an MTT assay under various conditions. THP-1 cells were cultured and seeded in a 96-well plate at a density of 1 × 10^5^ cells per well. Cells were divided into four groups; we used LPS (100 ng/mL LPS) (Thermo Fisher Scientific, Waltham, MA, USA) to activate the cells and improve the evaluation of the MTT assay: (1) LPS-stimulated control, (2) Transfection vehicle + LPS-stimulated, (3) HTT 90-5 + Stimulated by LPS (Thermo Fisher Scientific, Waltham, MA, USA), (4) and unstimulated control. The transfections were carried out using the appropriate transfection reagent according to the manufacturer’s protocol, followed by stimulation with LPS. After 24 h of incubation at 37 °C with 5% CO_2_, 10 µL of the MTT reagent (5 mg/mL) was added to each well and incubated for 4 h. Formazan crystals were solubilized using Isopropanol, and absorbance was measured at 595 nm using a microplate reader. Cell viability was calculated relative to the unstimulated control, and the results were statistically analyzed to determine the significance of the observed effects.

### 2.6. Real-Time PCR Measuring of Huntingtin Expression

RNA was extracted from cells grown in a six-well plate using Trizol reagent (Thermo Fisher Scientific, Waltham, MA, USA) according to the manufacturer’s protocol. The concentration and quality of the RNA were assessed using a Nanophotometer (Implen, Munich, Germany); the RNA purity was evaluated using the 260/230 and 260/280 ratios.

Subsequently, 1 μg of total RNA was transcribed to complementary DNA (cDNA) using the ImProm Reverse Transcription System (Promega Corporation, Madison, WI, USA) with oligo (dT) Primers. Subsequently, for the Huntingtin mRNA expression, the RT-PCR was performed using primers designed for amplifying the mRNA Huntingtin gene; these primers were designed with the NCBI primer software Version 2.5.0 (National Center for Biotechnology Information, Bethesda, MD, USA, Available at: https://www.ncbi.nlm.nih.gov/tools/primer-blast/, accessed on 11 April 2024), and the housekeeping gene was HPRT1, like the internal control. The sequences used were for Huntingtin expression, the forward primer was GCTGCACCGACCAAAGAAAG and the reverse primer was GTTCCATAGCGATGCCCAGA; for HPRT1, the forward primer was GACCAGTCAACAGGGGACAT and the reverse primer was GCTTGCGACCTTGACCATCT. We used SYBR Green PCR Master Mix (Thermo Fisher Scientific, Waltham, MA, USA), according to the manufacturer instructions. The reactions were run on a real-time PCR system under the following conditions: initial denaturation at 95 °C for 2 min, followed by 40 cycles of denaturation at 95 °C for 15 s and annealing/extension at 60 °C for 1 min. A melting curve analysis was included at the end of the run to confirm specificity.

The relative expression levels were calculated using the 2^−ΔCt^ method, where ΔCt is the difference between the Ct values of the target gene and the housekeeping.

### 2.7. Statistical Analysis

Data were expressed as the mean + standard error of the mean (SEM). Statistical comparisons between groups were made using one-way ANOVA followed by Tukey’s post hoc test, with *p* < 0.05 considered statistically significant compared to the control group.

## 3. Results

The patient was selected based on the inclusion and exclusion criteria mentioned in the methodology (Table 1). The selected patient was a 36-year-old female, dependent on her primary caregiver. The patient has 54 CAG repeats and was diagnosed in 2013; by the time of the sample collection, ten years had passed.

The synthesized antisense sequence, HTT 90-5, is as follows: CdTG-CdTG-CdTG-CdTG-CdTG-CdTG-CdTG-CdTG-CdTG-CdTG-CdTG-CdTG-CdTG-CdTG-CdTG-CdTG-CdTG-CdTG-CdTG-CdTG-CdTG-CdTG-CdTG-CdTG-CdTG-CdTG-CdTG-CdTG-CdTG-CdTG-GAAGG.

Cytosine (C), Adenine (A), Guanine (G).

**Figure 1:**
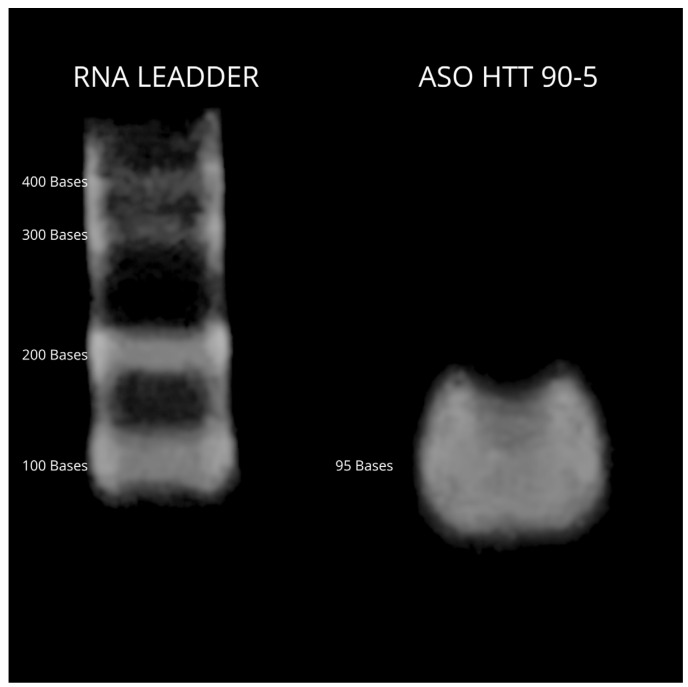
Validation of ASO correct synthesis.

This sequence has the following physicochemical characteristics: length, 95 nucleotides; GC content, 66.35%; melting temperature, 82.2 °C; molecular weight, 29,229.7 g/mole; extinction coefficient, 782,800 L (Mol*cm). A synthesis yield of fifty microliters of the sequence was obtained with a concentration of 2983 ng/μL, which was sufficient for the in vitro evaluation of the sequence. The length of the sequence was validated through a 2% Agarose gel (Figure 1), where a single band corresponding to the synthesized sequence was observed, confirming the expected molecular weight. No byproducts or other lengths were detected, indicating a successful synthesis.

**Figure 2:**
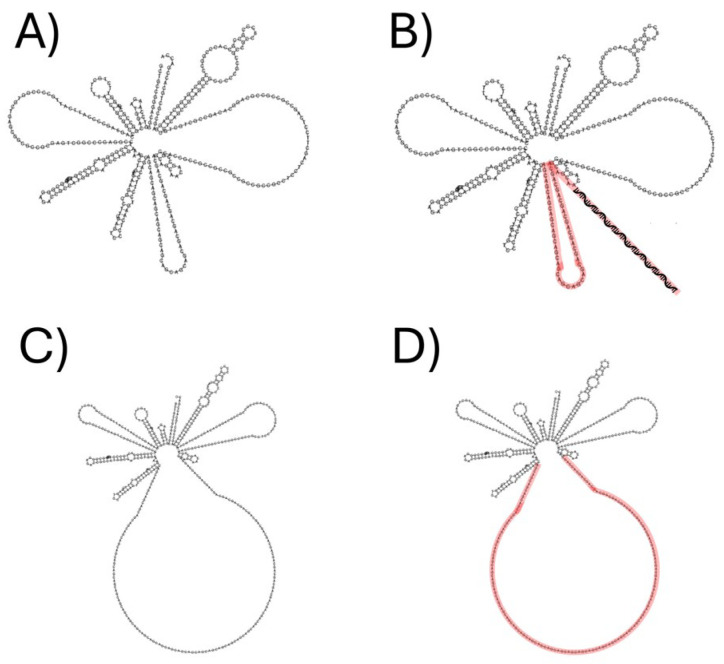
Secondary structure analysis of normal Huntingtin mRNA exon 1 (NCBI NM_001388492.1) and mRNA with over 30 CAG repeats. (**A**) Secondary structure of wild-type exon 1 Huntingtin (NCBI NM_001388492.1). (**B**) Secondary structure with the incomplete hybridization predicted between wild-type exon 1 Huntingtin and ASO HTT 90-5, the red color highlights the antisense sequence. (**C**) mRNA secondary structure of the patients >30 CAG repeats sequence predicted by RNAfold with total hybridization. (**D**) Complete hybridization between the mRNA secondary structure of the patients >30 CAG repeats sequence and ASO HTT 90-5, the red color highlights the antisense sequence.

**Figure 3:**
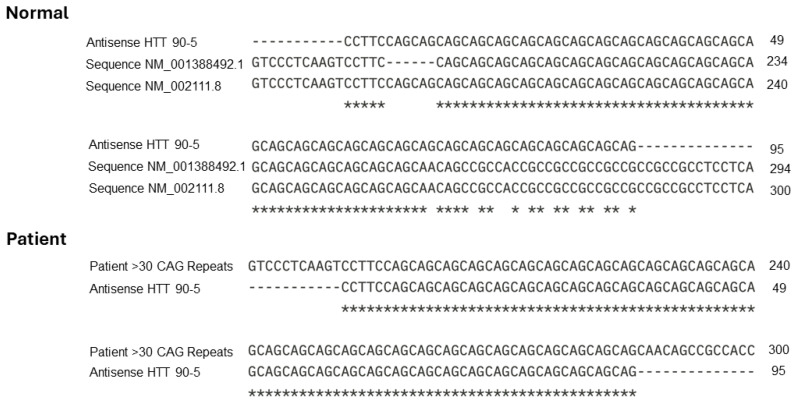
BLAST alignment of the antisense sequence ASO HTT 90-5 with the reference mRNA sequences Homo sapiens huntingtin transcript variant 1 (NCBI NM_001388492.1) and transcript variant 2 (NCBI NM_002111.8), as well as a sequence containing more than 30 CAG repeats, the asterisks indicate the nucleotides where homology between the sequences occurs.

Secondary structure prediction using RNAfold (Figure 2) showed that the antisense sequence hybridizes with the CAG repeat region of the Huntingtin mRNA within a loop structure formed between positions 51 nt and 146 nt of the coding sequence. This region’s secondary structure indicates that it is susceptible to silencing due to the loop conformation, allowing for efficient antisense hybridization. The alignment between the normal and mutated sequences (Figure 3) further confirms the targeted region. 

The alignment highlights the regions of homology between the antisense oligonucleotide and the expanded CAG repeat region, as well as the reference sequence. This comparation is important for evaluating the specificity of the antisense oligonucleotide for targeting the pathogenic CAG repeat expansion while ensuring minimal interaction with the normal allele.

Table 2 summarizes the thermodynamic and positional parameters of ASO HTT90-5 interactions with mutant Huntingtin mRNA and transcript variants. It highlights the strong specificity of ASO HTT90-5 for the pathological target, supporting its potential for allele-specific silencing.

Table 3 summarizes the sequences, molecular weights, melting temperatures and µg/OD values for the oligonucleotides used to measure HTT and HPRT1 expression.

**Figure 4:**
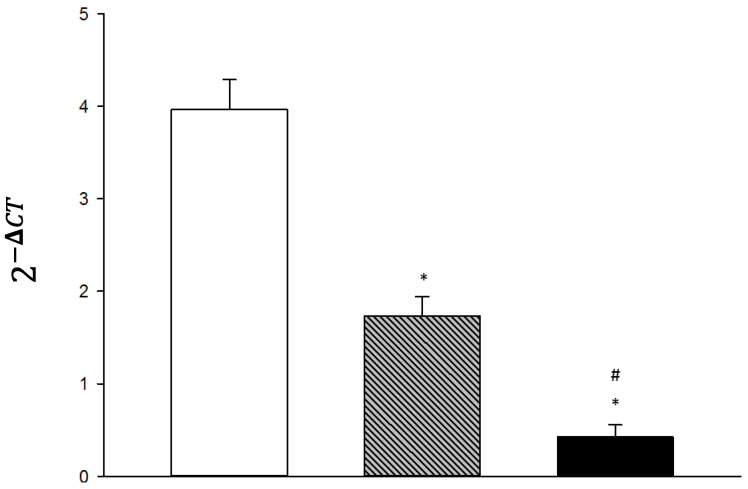
Relative expression of Huntingtin mRNA in primary culture leukocytes 72 h after in vitro treatment, * *p* < 0.05 vs. Control; # *p* < 0.05 vs. Vehicle.

In the primary leukocyte of the patient, a significant reduction in Huntingtin expression was observed after 72 h of treatment with ASO HTT 90-5, comparing both groups, the control and the lipofectamine (Thermo Fisher Scientific, Waltham, MA, USA) Vehicle group (Figure 4). Additionally, a significant decrease in gene expression was observed in the ASO HTT 90-5 group compared to that of the transfection vehicle group: * *p* < 0.05 vs. Control; # *p* < 0.05 vs. Vehicle.

**Figure 5:**
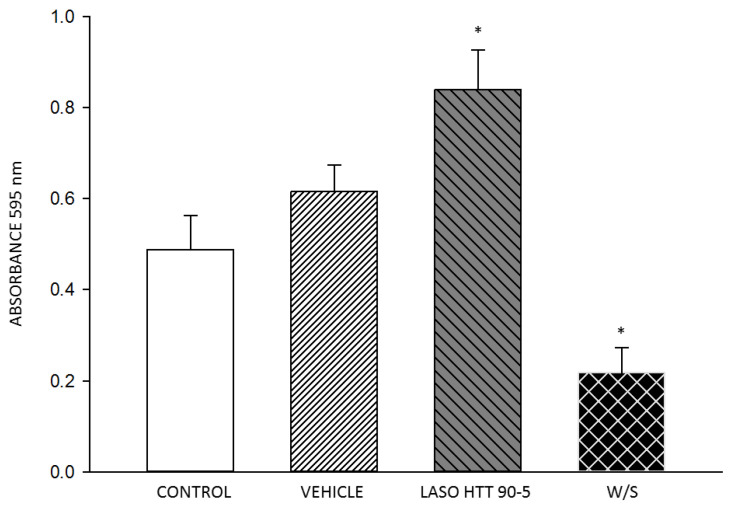
Absorbance measurement from the MTT assay for cell viability. * *p* < 0.05 vs. Control.

An MTT assay was performed to evaluate cell viability and potential toxicity. The results (Figure 5) indicate that the mitochondrial activity in unstimulated cells was low, making it difficult to reliably and reproducibly confirm. For this reason, cells were stimulated with LPS, which increases mitochondrial activity. We focused on LPS-stimulated cells, which provided consistent results, and we observed no significant changes in viability between the control and vehicle groups. However, in the HTT 90-5 ASO-treated group, there was a significant increase indicating an increase in metabolic activity.

Table 4 lists the sequences’ lengths and off-target analysis for the antisense oligonucleotide HTT 90-5, and its possible incomplete hybridizations were analyzed.

Table 5 presents the immunomodulatory scores for different segments of the analyzed sequence.

## 4. Discussion

The selection of Huntington’s Disease patients was based on heterozygosity for the Huntingtin gene, which is an important characteristic for our study. This is relevant because the hybridization of our antisense sequence strands specifically targets Huntingtin CAG repeats. Antisense Oligonucleotides (ASOs) that bind CUG RNA have been beneficial in DM1 models by altering the protein interactions or metabolism of the toxic RNA [28]. This approach could generate greater selectivity towards the mutated Huntingtin sequence [28,29]. Additionally, this may stabilize CTG repeats at sub-pathogenic lengths, as observed in other conditions involving repeats instability [30].

CTG repeat expansions or contractions are frequent events in human cells; ASOs may provide a targeted approach to modulate instability [28]. One therapeutic approach has focused on CAG-repeat antisense oligonucleotides (ASOs) designed to bind CUG repeat RNA, blocking RNA-protein interactions through steric inhibition [31]. Antisense oligonucleotides (ASOs) have been effective in reversing RNA toxicity in myotonic dystrophy by reducing mutant RNA and correcting splicing defects. Applying this approach to Huntington’s disease, our HTT 90-5 ASO could potentially mitigate disease progression by disrupting the toxic RNA interactions, as seen in similar antisense therapies for other repeat expansion disorders [32].

Our results have shown that leukocytes express Huntingtin, consistent with previous studies that have also identified its expression in these cells [33]. Furthermore, it has been observed that the increase in Huntingtin expression in leukocytes has been associated with the disease progression and atrophy of the caudate nucleus, suggesting a possible role as a biomarker of disease progression [34]. Multiple studies have utilized leukocytes to evaluate antisense efficacy, confirming that these cells could be transfected and are useful for antisense evaluation [34]. This background could support the use of leukocytes as a model for evaluation in the selected patient.

Other groups have addressed the problem of allele selectivity by directing the antisense or siRNAs to SNPs with a greater prevalence in certain phenotypes of mutated alleles [35]. We propose directing the sequences towards the CAG region, which differs significantly between normal and mutated alleles in HD [32,36]. This work is an initial study step to confirm the effectiveness of the antisense in reducing expression and generating antisense as a therapeutic novel strategy. In the future, we aim to explore the mechanisms underlying this effect and about the specificity of the sequence for the mutated allele.

The design of the long antisense oligonucleotide is based on previous evidence suggesting that long non-coding RNA sequences can modulate gene expression, act as expression regulators and function as RNA silencers [37]. These kinds of sequences have been shown to repress several proteins and even modulate the expression of entire chromosomes [38]. This occurs through mechanisms that include the recruitment of proteins that increase or decrease gene expression [39], interference with RNA binding to the ribosome, RNA degradation and other mechanisms that include direct binding to DNA or RNA sequences, thereby generating the inhibition of transcription. They also serve as sponges for miRNAs and regulate the alternative splicing of some genes [40].

ASO HTT 90-5 was specifically designed to hybridize with sequences containing more than 30 CAG repeats, thus distinguishing pathological from normal alleles. This design is based on the statistical difference in the distribution of CAG repeats between affected patients. Similar antisense studies have been conducted for diseases such as myotonic dystrophy type 1 [28]. In a previous study by Dr. Masayuki (2011), a significant decrease in the expression of Huntingtin was achieved using an antisense oligonucleotide targeting the CAG cluster [29]. In our case, we designed a long RNA antisense of 95 nucleotides which cover 30 CAG repeats in addition to 5 nucleotides after these repeats in the gene to increase specificity and avoid the off-targeting of genes or sequences that had CAG repeats in other regions. To address the overlap in CAG, repeat lengths between wild-type and mutant alleles, it is important to note that most of the population carries *Huntingtin* alleles with fewer than 30 CAG repeats, reducing the risk of off-target effects. However, for individuals with normal alleles containing >30 repeats, longer antisense oligonucleotides (ASOs) with enhanced specificity are needed. While this approach could further mitigate off-target interactions, a major limitation is the reduced yield and efficiency during the synthesis of longer ASOs. This issue underscores the need for the optimization of synthesis methods and the incorporation of chemical modifications to improve both scalability and therapeutic applicability. Additionally, the evaluation of allele-specific silencing in vivo remains a critical next step to refine these therapeutic strategies [11].

The specificity of ASO HTT 90-5 was corroborated through bioinformatic analysis, using methods that we have reported previously to ensure no significant off-targets [41]. This analysis is crucial to minimizing adverse effects. The full-length antisense oligonucleotide antisense blast did not reveal significant similarities to other sequences, consistent with the high specificity expected from longer oligonucleotides. However, when we reduce the length of the sequence to 20 nucleotides in the silico analysis to explore the potential off-target in a probable non-complete hybridization, one off-target was identified, the myocyte factor 2A gene, with 95% query coverage in the 20 nucleotides analysis. MEF2 is a key transcription factor which regulates the expression of genes involved in muscle development and differentiation; this factor helps to control bone development [42]. The off target identified represents only a potential interaction with a short 20-nucleotide fragment; the possible implications of silencing MEF2A could include unwanted effects on muscular or neuronal function. Although this off-target interaction is predicted for a short 20-nucleotide hybridization, the full-length 95-nucleotide sequence is unlikely to have sufficient complementary to silence the MEF2A, minimizing the risk of this interference [43].

On the other hand, we used Version 3.4.1, IntaRNA software (https://rna.informatik.uni-freiburg.de/IntaRNA/Input.jsp;jsessionid=EB146E810942D182186E7DE611E37ADC, accessed on 14 November 2024) to demonstrate the high specificity of ASO HTT90-5 for targeting mutant *Huntingtin* mRNA with >30 CAG repeats. The interaction with the sequence >30 CAG exhibited the lowest total energy (−154.92 kcal/mol), indicating strong and stable binding. In contrast, interactions with transcript wild-type variants showed less favorable energetics (−116.53 and −107.63 kcal/mol), suggesting reduced potential for off-target effects in persons with <30 CAG repeats in his Huntingtin sequence alleles. Additionally, the critical seed region (positions 46–52 in the target) was consistently involved across all interactions, ensuring the reliable initiation of hybridization. These findings, summarized in Table 2, highlight the potential of ASO HTT90-5 as a precise therapeutic agent capable of effectively targeting mutant Huntingtin mRNA while minimizing interactions with wild-type sequences with <30 CAG repeats [44].

The immunogenicity analysis of the oligonucleotide HTT 90-5 showed scores between 4.7 and 5.9, indicating a moderate potential for immune activation. This suggests the possibility of triggering immune responses which could impact the safety of the sequences [45]. However, the MTT assay did not show a reduction in cell viability, indicating that ASO HTT 90-5 does not induce significant cytotoxicity in leukocytes (Figure 5). Additional studies are required to determine whether the ASO triggers inflammatory responses. Based on this, our antisense was modified by substituting uracil with thymine to reduce immunogenicity, as previously reported. Additionally, this modification can increase enzyme resistance and potentially extend the ASO’s half-life; this was not directly measured in our study but has been documented in the literature [46]. The secondary structure of the hybridization site was predicted to form a favorable loop, probably allowing for efficient molecular interactions [47].

These findings support the therapeutic potential of ASO HTT 90-5 in the treatment of Huntington’s disease and provide a basis for the future development of antisense therapies targeting other genetic disorders using similar approaches. Future research should explore intranasal administration as a delivery method for ASO HTT90-5. Intranasal delivery offers several advantages, including the ability to bypass the blood–brain barrier and achieve localized delivery to CNS regions affected by Huntington’s disease. While this method has shown promise in preclinical models for other oligonucleotide therapies, further studies are needed to evaluate its formulation, stability and efficacy specifically for ASO HTT90-5. Additionally, lipofectamine has demonstrated success as a transfection agent in primary cultured human cells under controlled in vitro conditions, highlighting its potential applicability in specific scenarios. Nonetheless, alternative delivery systems, such as nanoparticles, lipid-based carriers and ligand-conjugated methods (e.g., GalNAc), should be prioritized due to their improved biocompatibility and lower-toxicity profiles [23,24,48,49,50].

## 5. Conclusions

The antisense oligonucleotide HTT 90-5, designed to target the CAG repeat cluster in the Huntingtin gene, effectively reduced mutant Huntingtin expression in patient-derived primary leukocyte cultures without compromising cell viability, highlighting its potential as a therapeutic candidate for Huntington’s Disease. Bioinformatic analyses suggest probable specificity for pathogenic CAG repeats while minimizing interactions with wild-type alleles, though experimental validation is needed to confirm this and strengthen future findings. The use of leukocytes as proxy models demonstrates the feasibility of alternative cellular approaches for initial evaluations. Future directions include optimizing delivery methods, such as nanoparticles, ligand-conjugated carriers and intranasal administration, to enhance CNS targeting and therapeutic applicability. These findings lay the groundwork for advancing HTT 90-5 and similar personalized therapies for Huntington’s Disease.

## Figures and Tables

**Table 1:** Patient Data.

Attribute	Details
Gender	Female
Age	36 years
Children	None
Caregiver Dependence	Yes
CAG Repetitions	54 (mutant allele)/19 (wild-type allele)
Year of Diagnosis	2013
Family History	Paternal grandmother, paternal aunt, father
Clinical Phenotype	Choreic phenotype
UHDRS Motor Score	16/28 (Moderate to Severe)
UHDRS Cognition Score	2/12 (Mild)
UHDRS Behavior Score	6–8/12 (Moderate)
UHDRS Functionality Score	7–8/8 (Severe)
UHDRS Total Score	31–34/60 (Moderate to Severe)

**Table 2:** RNA Interaction Analysis of ASO HTT90-5 with Different Huntingtin Transcripts.

Target RNA	Energy (kcal/mol)	Hybridization Energy (kcal/mol)	Unfolding Energy—Target (kcal/mol)	Unfolding Energy—Query (kcal/mol)	Position—Target	Position—Query	Position Seed—Target	Position Seed—Query
Huntingtin transcript > 30 CAG repeats	−154.92	−232.98	37.28	40.78	46—140	1—95	46—52	89—95
Huntingtin transcript variant 2	−116.53	−189.58	32.27	40.78	46—138	3—95	46—52	89—95
Huntingtin transcript variant 1	−107.63	−181.6	33.19	40.78	46—141	3—95	46—52	89—95

**Table 3:** Huntingtin and HPRT Primers.

Oligo Name	Sequence	# of Bases	Molecular Weight (MW)	Melting Temperature (Tm, °C)	µg/OD
HTT expression F	GCTGCACCGACCAAAGAAAG	20	6129	57	30.55
HTT expression R	GTTCCATAGCGATGCCCAGA	20	6102	57.2	31.45
HPRT1 HOMO F	GACCAGTCAACAGGGGACAT	20	6160.1	56.9	30.26
HPRT1 HOMO R	GCTTGCGACCTTGACCATCT	20	6044	57.8	34.22

**Table 4:** Off-target analysis.

Number of Nucleotides	Sequence	Off-Targets	Query Cover
95 nts	CTGCTGCTGCTGCTGCTGCTGCTGCTGCTGCTGCTGCTGCTGCTGCTGCTGCTGCTGCTGCTGCTGCTGCTGCTGCTGCTGCTGCTGCTGGAAGG	No significant similarity	NA
80 nts	CTGCTGCTGCTGCTGCTGCTGCTGCTGCTGCTGCTGCTGCTGCTGCTGCTGCTGCTGCTGCTGCTGCTGCTGCTGGAAGG	No significant similarity	NA
65 nts	CTGCTGCTGCTGCTGCTGCTGCTGCTGCTGCTGCTGCTGCTGCTGCTGCTGCTGCTGCTGGAAGG	No significant similarity	NA
50 nts	CTGCTGCTGCTGCTGCTGCTGCTGCTGCTGCTGCTGCTGCTGCTGGAAGG	No significant similarity	NA
35 nts	CTGCTGCTGCTGCTGCTGCTGCTGCTGCTGGAAGG	No significant similarity	NA
20 nts	CTGCTGCTGCTGCTGGAAGG	Myocyte enhancer factor 2A (MEF2A)	95%

**Table 5:** Immunogenicity Analysis.

Range of Positions	Sequence	Immunomodulatory Score
1–64	CUGCUGCUGCUGCUGCUGCUGCUGCUGCUG	5.8
65–66	UGCCUGCUGCUGCUGCUGCUGGAAG	5.4
67	GCUGCUGCUGCUGCUGCUGCUGGAAGG	5.2
68	CUGCUGCUGCUGCUGCUGCUGGAA	4.7
69	UGCCUGCUGCUGCUGCUGCUGGAA	4.9

## Data Availability

The data are contained in the article.
